# Extrastriatal ^123^I-FP-CIT SPECT impairment in Parkinson’s disease – the PPMI cohort

**DOI:** 10.1186/s12883-020-01777-2

**Published:** 2020-05-16

**Authors:** Nicolas Nicastro, Valentina Garibotto, Pierre R. Burkhard

**Affiliations:** 1 Department of Psychiatry, University of Cambridge, Cambridge, UK; 2 Division of Neurology, Department of Clinical Neurosciences, Geneva University Hospitals, 4, rue G. Perret-Gentil, 1205 Geneva, Switzerland; 3 Department of Nuclear Medicine, Geneva University Hospitals, Geneva, Switzerland; 4 Faculty of Medicine, University of Geneva, Geneva, Switzerland

**Keywords:** Parkinson’s disease, Dopamine, Serotonin, SPECT

## Abstract

**Background:**

Neuropathological data and nuclear medicine imaging show extensive serotonergic impairment in Parkinson’s disease (PD). We undertook a case-controlled analysis of ^123^I-FP-CIT SPECT images to measure extrastriatal serotonergic transporters (SERT) in PD using the Parkinson’s Progression Markers Initiative (PPMI) cohort.

**Methods:**

We included all PD (*n* = 154) and Control subjects (*n* = 62) with available ^123^I-FP-CIT SPECT imaging and high-resolution T1-weighted MRI for coregistration (PD: mean age 61.6 years, 62% male, disease duration 26 months, MDS-UPDRS III score 22). ^123^I-FP-CIT SPECT images were processed with PETPVE12 using an exploratory voxel-wise analysis including partial-volume effect correction. Linear regressions were performed in the PD group to assess correlations between region of interest ^123^I-FP-CIT uptake and clinical motor and non-motor impairment.

**Results:**

Compared to Controls, PD exhibited an uptake reduction in bilateral caudate nucleus, putamen, insula, amygdala and right pallidum (family-wise error (FWE)-corrected *p* <  0.05). While lower putaminal uptake on the contralateral side to clinically more affected side was associated with higher MDS-UPDRS III score (*p* = 0.022), we found a trend association between higher geriatric depression scale and lower pallidum uptake (*p* = 0.09). Higher SCOPA-AUT gastrointestinal subscore was associated with lower uptake in mean putamen and caudate nucleus (*p* = 0.01 to 0.03), whereas urological subscore was inversely correlated with mean caudate nucleus, putamen, and pallidum uptake (*p* = 0.002 to 0.03). REM sleep behaviour disorder screening questionnaire was associated with lower ^123^I-FP-CIT binding in caudate nucleus, putamen and pallidum (all *p* <  0.05). No significant association was found for Montreal Cognitive Assessment (all *p* > 0.45) or excessive daytime sleepiness (all *p* > 0.29).

**Conclusions:**

In addition to the well-established striatal deficit, this study provides evidence of a major extrastriatal ^123^I-FP-CIT impairment, and therefore of an altered serotonergic transmission in early PD.

## Background

^123^I-N-ω-fluoropropyl-2β-carbomethoxy-3β-(4-iodophenyl) nortropane (^123^I-FP-CIT) is the most widely used single photon emission computed tomography (SPECT) ligand to assess the integrity of presynaptic nigrostriatal pathways [1]. Thus far, little attention has been paid to extrastriatal binding of ^123^I-FP-CIT which mainly (70%) derives from serotonergic transporters (SERT) density [2]. Serotonin (5-hydroxy-tryptamine, 5-HT) regulates many higher brain functions including cognition, mood and motor behaviour – all being impaired in degenerative parkinsonisms. 5-HT is synthesized in the raphe nuclei of the brainstem which project rostrally to the cortex, thalamus and basal ganglia and caudally to the spinal cord [3]. Evidence from post-mortem and in vivo ^11^C-3-amino-4-(2-dimethylaminomethyl-phenylsulfanyl) benzonitrile (^11^C-DASB) positron emission tomography (PET) imaging has demonstrated serotonergic cell loss in degenerative forms of parkinsonism, especially in Parkinson’s disease (PD) and Progressive Supranuclear Palsy (PSP) [4–6]. ^123^I-FP-CIT SERT concentration has been shown to be higher in females and to decline with age [7], and reduced uptake has been observed for PD in frontal areas, thalamus, amygdala and insula [8, 9], confirming ^11^C-DASB PET findings.

Using the Parkinson’s Progression Markers Initiative (PPMI) database [10], we here propose a case-controlled extrastriatal ^123^I-FP-CIT assessment of early PD subjects. In addition, we performed correlation analyses of striatal and extrastriatal significant regions of interest (ROI) uptake with clinical motor and non-motor scales, i.e. Movement Disorders Society (MDS)-Unified Parkinson’s Disease Rating Scale (UPDRS) part III at baseline and at 1-year follow-up [11], Geriatric Depression Scale (GDS) [12], Montreal Cognitive Assessment (MoCA) [13], SCale for Outcome in PArkinson’s disease – AUTonomic dysfunction (SCOPA-AUT) [14], Epworth Sleepiness Scale (ESS) [15] and REM-sleep behaviour screening questionnaire (RBDSQ) [16].

## Methods

### Participants

As of 4th February 2019, 423 PD and 196 Controls (CTL) were enrolled in the PPMI study. For the present work, we included all subjects who had both ^123^I-FP-CIT SPECT imaging and high-resolution 3 T T1-weighted magnetization-prepared rapid gradient echo (MPRAGE) MRI performed within 3 months. In addition, inclusion was limited to individuals aged over 40 years to ensure that only sporadic rather than genetic cases were taken into consideration. We also collected clinical data from the PPMI database, i.e. total scores of the MDS-UPDRS III scale (off-state, ≥6 h after dopaminergic treatment discontinuation) at baseline (within 3 months of scans) and at 1-year follow-up, as well as Hoehn and Yahr stage [17]. Non-motor impairment at baseline was assessed using measures of cognition (MoCA) [13], depression (GDS) [12], dysautonomia with SCOPA-AUT scale [14], including gastrointestinal (SCOPA-GI, 7 items) and urinary (SCOPA-URO, 6 items) subscores, as well as excessive daytime sleepiness (ESS) [15] and REM-sleep behaviour disorder (RBDSQ) [16].

### MRI imaging

All patients were scanned on a 3 T Siemens Magnetom TRIO Tim MRI scanner (Siemens Medical Solutions, Erlangen, Germany) using a 32-channel head coil. MPRAGE structural T1-weighted images were acquired with the following parameters: TR = 2300 ms, TE = 3 ms, flip angle = 9°, 256x256x240 mm matrix and voxel size = 1 mm3 (isotropic).

### SPECT imaging acquisition, reconstruction and preprocessing

Reconstructed and attenuation-corrected ^123^I-FP-CIT SPECT imaging data was downloaded from the PPMI website. Images were acquired on a Siemens or General Electric SPECT tomographs, 3-4 h after ^123^I-FP-CIT injection. The imaging protocol for the PPMI scans is documented in http://www.ppmi-info.org/wp-content/uploads/2013/02/PPMI-Protocol-AM5-Final-27Nov2012v6-2.pdf.

Preprocessing of SPECT brain images was performed using Statistical Parametric Mapping (SPM12, Wellcome Trust Centre for Neuroimaging, London, UK, https://www.fil.ion.ucl.ac.uk/spm/), running in MATLAB R2018b Version 9.5.0 (MathWorks Inc., Sherborn, MA, USA). SPECT images were coregistered to each patient’s own MPRAGE structural MRI, partial-volume-effect-corrected with the PETPVE12 toolbox running on SPM [18] after segmenting the MRI into grey matter, white matter and CSF tissue compartments using tissue probability maps. Resulting SPECT images were then intensity normalised using the occipital lobe as the reference region and warped into Montreal Neurological Institute (MNI) standard space. We performed a two-sample t-test (PD vs CTL group), with age, sex, antidepressant medication and medical centre as covariates. T-maps contrasts were obtained by comparing groups with family-wise error (FWE)-corrected *p* <  0.05, limiting the results to the expected cluster voxel size according to SPM.

### ROI-based analyses

**Fig. 1:**
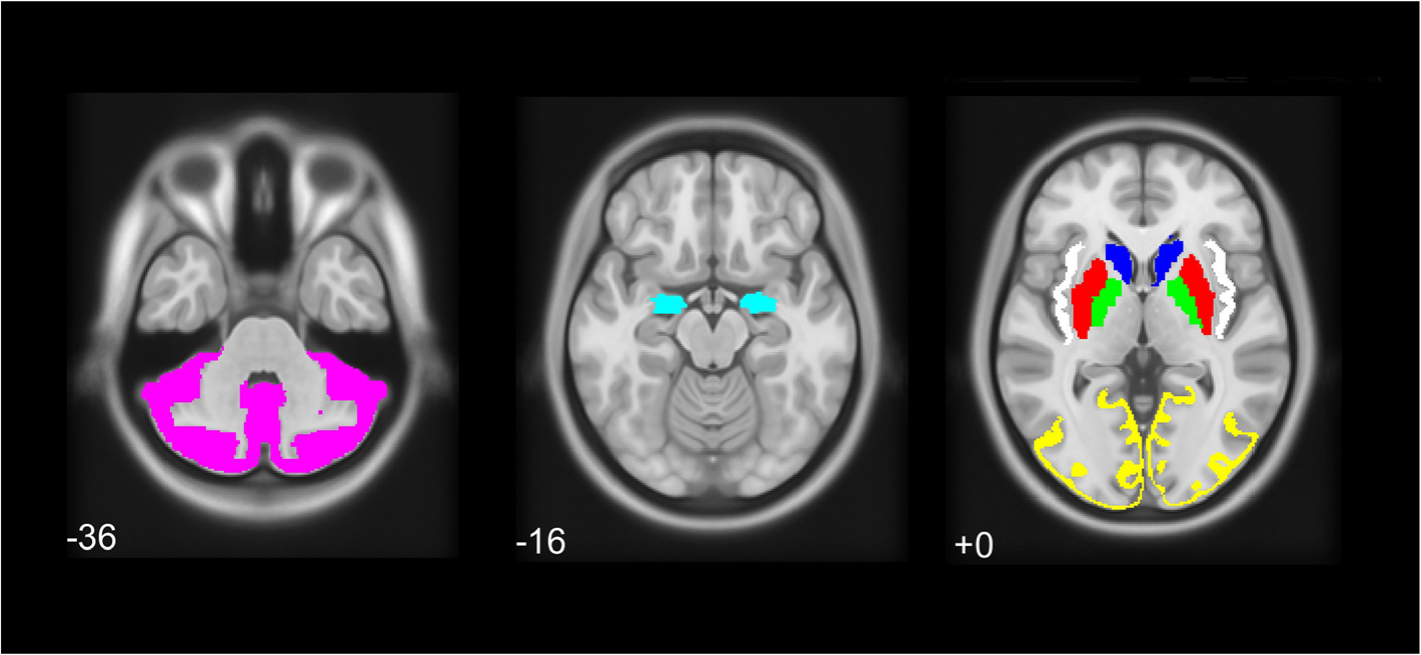
Regions of interest used for the present study, extracted with the Desikan-Killiany atlas: (left) cerebellum grey matter (*magenta*), (center) amygdala (*cyan*), (right) caudate nucleus (blue), putamen (*red*), pallidum (*green*), insula (*white*) and occipital lobe grey matter (*yellow*). Numbers represent Z-axis coordinates in Montreal Neurological Institute space

Uptake extraction of the significant striatal and extrastriatal ROIs found in the voxel-wise analysis was performed with PETPVE12 [18] for correlation with clinical scales. After SPECT/MRI coregistration and MRI segmentation described above, we proceeded to ROI uptake extraction using geometric transfer matrix (GTM), including partial volume effect (PVE) correction. SPECT images were smoothed using an 8 mm full width at half-maximum (FWHM) Gaussian kernel. The Desikan-Killiany was used for ROI uptake extraction [19] (Fig. 1). Semiquantitative values were obtained using the occipital lobe as the reference (REF) for DAT-rich ROIs (caudate nucleus, putamen and pallidum) or cerebellar grey matter for SERT-rich regions (amygdala and insula) [20] using the specific/non-specific binding ratio (SBR) as SBR = (Uptake_ROI_ – Uptake_REF_)/Uptake_REF_.

### Statistical analysis

Statistical analyses were performed with Stata Version 14.2 software (College Station, TX). Continuous variables were assessed for normality with plotted histograms and Shapiro-Wilk test. We used t-test or Mann-Whitney U test (MWU) for continuous variables with a normal and non-normal distribution, respectively. χ^2^ test was performed for categorical variables.

Correlation analyses were performed for PD using univariate linear regressions between clinical motor/non-motor scales and ROIs with significantly reduced uptake in PD. Between-ROI correlations were also performed using the same covariates. When assessing a correlation with a motor scale (MDS-UPDRS III), we used age, sex, antidepressant medication and centre as covariates and striatal ROIs uptake on the contralateral side to clinically more affected side according to MDS-UPDRS III lateralising items. These included appendicular rigidity, all items of bradykinesia (finger tapping, hand movements, pronation-supination movements of hand, toe tapping, leg agility), postural and kinetic tremor of hands. Axial items included speech, facial expression, rigidity of the neck, arising from chair, gait, freezing of gait, postural stability and posture.

For non-motor scales (GDS, MoCA, SCOPA subscores, ESS and RBDSQ), MDS-UPDRS III score was added as an additional covariate to adjust for motor severity and the mean (average of left and right) ROI uptake was used as the independent variable.

## Results

### Patients demographics

**Fig. 2:**
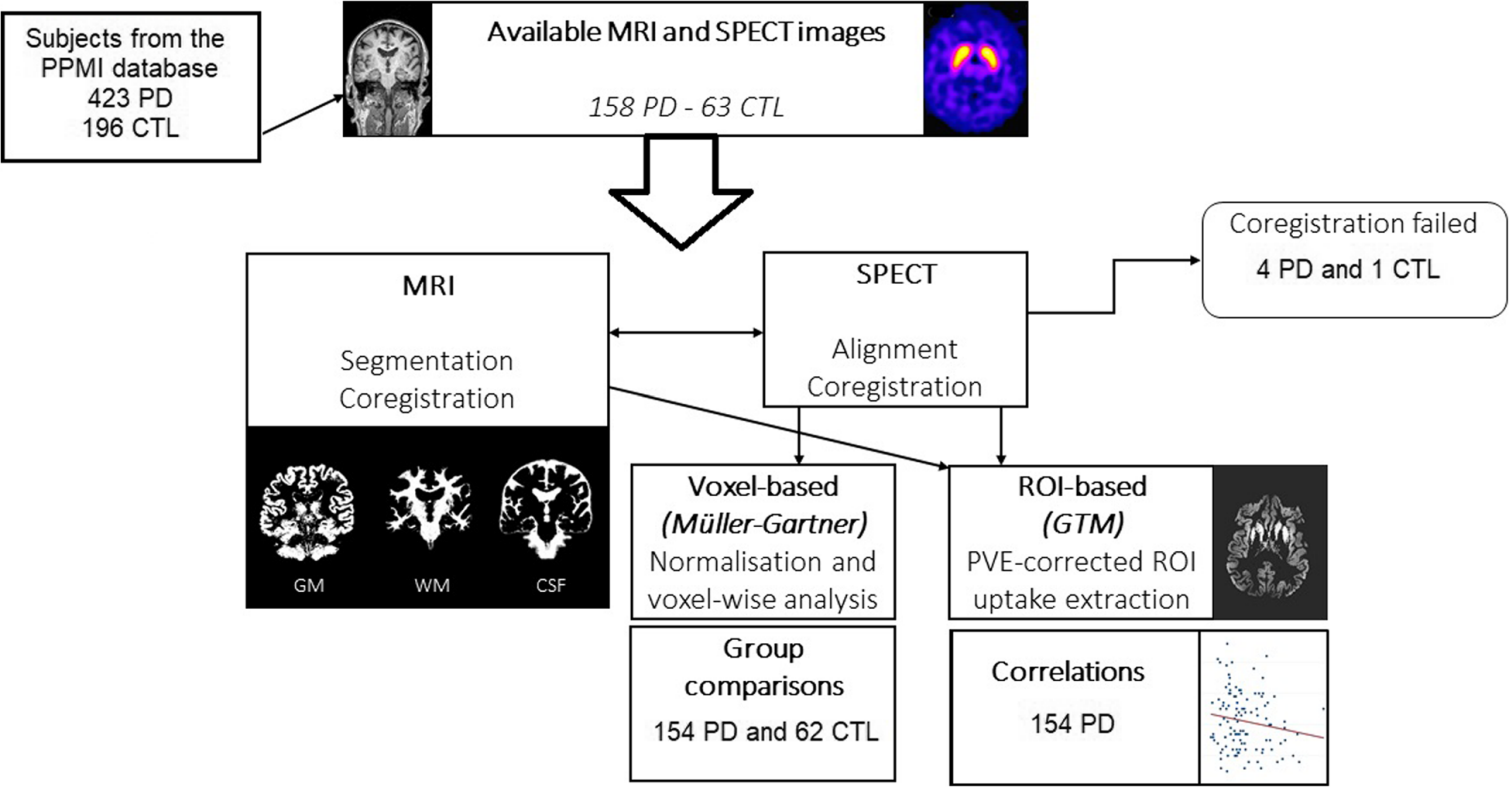
inclusion process of the study

According to our inclusion criteria (subjects aged ≥40 years, available SPECT and MPRAGE MRI performed within 3 months interval), 158 early PD and 63 CTL from 12 different medical centres were eligible for the present work. During the image preprocessing, 4 PD and 1 CTL subjects were excluded because of technical issues related to SPECT/MRI coregistration. Eventually, 154 PD and 62 CTL were included for group comparisons. Demographics and inclusion flowchart are available in Table 1 and Fig. 2. Relative to Controls, PD patients had significantly higher scores for MDS-UPDRS III (including total score as well as axial and lateralising items subscores, all *p* <  0.0001, *MWU*), Hoehn and Yahr scale (p <  0.0001, *Chi-Squared test*), RBDSQ (*p* < 0.001, *MWU*), and SCOPA-AUT (incl. Total score, SCOPA-GI and SCOPA-URO subscores, all *p* < 0.05, *MWU*). No significant differences for depression (as measured with GDS), cognition (MOCA) and excessive daytime sleepiness (ESS) were observed (all *p* > 0.18, *MWU*).

**Table 1:** Demographics for the PD and CTL groups

	PD (*n* = 154)	CTL (*n* = 62)	*p*-value
Age (years)	61.6 ± 8.9 (40–82)	61.5 ± 10 (40–81)	0.87 §
Male distribution	61.7% (98/154)	61.5% (41/62)	0.73 #
Right-handedness	90.3% (139/154)	82.3% (51/62)	0.19 #
Disease duration (months)	25.5 ± 17.8 (2–77)	NA	–
MDS-UPDRS III at baseline	22.3 ± 9.7 (5–46)	0.5 ± 1.2 (0–8)	< 0.0001 §
*MDS-UPDRS III axial subscore*	4.0 ± 2.6 (0–12)	0.1 ± 0.3 (0–2)	< 0.0001 §
*MDS-UPDRS III right-sided items subscore*	7.9 ± 4.8 (0–20)	0.2 ± 0.5 (0–3)	< 0.0001 §
*MDS-UPDRS III left-sided items subscore*	7.7 ± 6.1 (0–23)	0.3 ± 0.6 (0–3)	< 0.0001 §
Hoehn-Yahr scale	1.5 ± 0.5 (1–2)	0 (0–0)	< 0.001 #
MOCA	27.4 ± 2.2 (20–30)	28.2 ± 1.2 (26–30)	0.19 §
GDS	5.3 ± 1.4 (1–11)	5.1 ± 1.2 (3–10)	0.39 §
SCOPA-AUT	10.5 ± 6.0 (0–40)	7.5 ± 4.5 (0–19)	0.0002 §
*SCOPA-AUT Gastrointestinal subscore*	2.3 ± 1.9 (0–11)	0.8 ± 1.0 (0–4)	< 0.0001 §
*SCOPA-AUT Urological subscore*	3.9 ± 2.5 (0–14)	3.2 ± 2.3 (0–12)	0.03 §
Epworth Sleepiness Scale	6.2 ± 4.0 (0–19)	5.9 ± 3.3 (0–15)	0.68 §
REM-sleep behavior disorder screening questionnaire	4.3 ± 2.6 (1–12)	2.9 ± 2.2 (0–10)	< 0.001 §

Baseline demographics of PD and CTL subjects included in the study. Statistical tests: Chi-square #, Mann-Whitney U test §. *NA* not applicable

### Voxel-based ^123^I-FP-CIT SPECT analyses

**Fig. 3:**
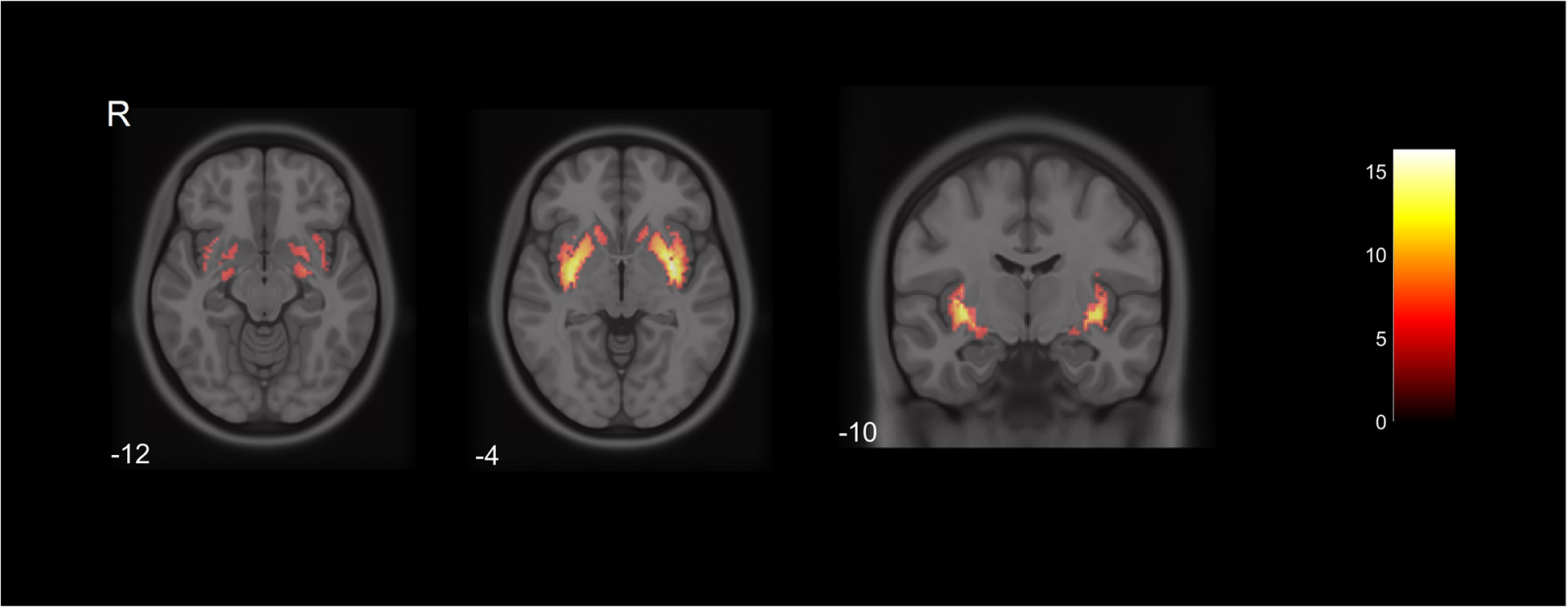
Axial and coronal slices of voxel-wise ^123^I-FP-CIT SPECT group comparisons between CTL and PD subjects. Cluster #1 *(left, center and right)* includes right insula, putamen, amygdala and pallidum, Cluster #2 *(left, center and right)* includes left insula, putamen and caudate nucleus and amygdala, Cluster #3 *(center)* includes right caudate nucleus (FWE-corrected *p* < 0.05). Color bar represents T-score. R = right. Numbers are Z-axis coordinates in MNI space

Compared to the CTL group, PD subjects showed decreased uptake in a first cluster (total 1109 *voxels* (vx), peak level T-score 16.3) including right insula, putamen, amygdala and pallidum. The second cluster (total 1195 *vx*, peak level T-score 14.4) included left insula, putamen and caudate nucleus and amygdala. A third smaller cluster (140 vx, peak level T-score 7.4) included right caudate nucleus (all FWE-corrected *p* < 0.05) (Fig. 3).

### Correlation analysis of clinical scales and ROIs ^123^I-FP-CIT uptake

ROI-based uptake values of striatal (caudate nucleus and putamen) and extrastriatal (pallidum, insula and amygdala) regions of interest are available in Table 2. Strong correlations were observed between caudate nucleus, putamen and pallidum ^123^I-FP-CIT uptake (all *p* < 0.001), while insula binding correlated with caudate nucleus and putamen binding (both *p* < 0.001) but not with pallidum (*p* = 0.83). Uptake in the amydala correlated with putamen (*p* = 0.004) and insula (*p* < 0.001), but not with caudate nucleus (*p* = 0.14) or pallidum (*p* = 0.20). Similar findings were observed when studying the subgroup of PD patients with GDS ≥ 5. However, when assessing only PD patients with mild cognitive impairment (MOCA ≤25, *n* = 30), we did not observe any significant correlation between striatal and extrastriatal, except for a trend association between putamen and amygdala ^123^I-FP-CIT binding (*p* = 0.09).

**Table 2:** Regional 123I-FP-CIT uptake values for the PD and Control groups

	PD (*n* = 154)	Controls (*n* = 62)	*p*val
Caudate nucleus	2.66 ± 0.95	3.60 ± 1.03	< 0.0001
Putamen	1.34 ± 0.46	2.54 ± 0.44	< 0.0001
Pallidum	1.51 ± 0.80	2.56 ± 0.56	< 0.0001
Insula	0.43 ± 0.16	0.64 ± 0.19	< 0.0001
Amygdala	0.52 ± 0.21	0.59 ± 0.19	0.01

Average (mean of left and right) ^123^I-FP-CIT uptake values for PD and Control groups for striatal and extrastriatal regions-of-interest. For comparative purposes, the same reference region (occipital lobe) was used for the present results. All group comparisons are performed using Mann-Whitney U test

Higher MDS-UPDRS III was associated with lower ^123^I-FP-CIT uptake in the putamen contralateral to clinically more affected side (*p* = 0.022, coefficient = − 9.2, 95% confidence interval (CI) -17 to − 1.4). No significant correlation was found between ROIs uptake and MoCA (all *p* > 0.45). We found a trend association between higher GDS and lower mean ^123^I-FP-CIT pallidal uptake (*p* = 0.08, coefficient − 0.65, 95% CI − 1.39 to 0.09). Higher SCOPA-GI correlated with lower mean putamen (*p* = 0.01, coefficient − 2.2, 95% CI − 3.9 to − 0.5) and caudate nucleus uptake (*p* = 0.034, coefficient − 1.48, 95% CI − 2.9 to − 0.1). Finally, higher SCOPA_URO subscore was associated with lower mean uptake in the caudate nucleus (*p* = 0.002, coefficient − 2.7, 95% CI − 4.3 to − 1.0), putamen (*p* = 0.013, coefficient − 2.7, 95% CI − 4.6 to − 0.6) and pallidum (*p* = 0.025, coefficient − 1.4, 95% CI − 2.7 to − 0.2).

No significant correlation was observed between ESS and ROIs ^123^I-FP-CIT uptake (all *p* > 0.29). However, we observed that – among PD participants – higher RBDSQ was associated with lower ^123^I-FP-CIT uptake in caudate nucleus, putamen and pallidum (all *p* < 0.05). All correlations are available in Table 3.

**Table 3:** Correlations between clinical motor and non-motor scales and regional 123I-FP-CIT uptake in PD

Score	Region of interest	Coefficient	pval	95% Confidence Interval
MDS-UPDRS III	PUT*	−9.2	**0.022**	[− 17; − 1.4]
	CN*	−4.4	0.44	[− 15.6; 6.8]
	PAL*	−1.46	0.67	[−8.1; 5.2]
	INS	− 3.4	0.22	[−8.9; 2.0]
	AMYG	−1.8	0.25	[−4.8; 1.2]
MOCA	PUT	−0.47	0.62	[−2.3; 1.4]
	CN	−0.26	0.74	[−1.8; 1.3]
	PAL	−0.1	0.85	[−1.2; 1]
	INS	−0.02	0.97	[−1.3; 1.2]
	AMYG	−0.26	0.46	[−1; 0.4]
GDS	PUT	0.54	0.39	[−0.7; 1.8]
	CN	−0.30	0.56	[−1.3; 0.7]
	PAL	−0.65	0.08	[−1.4; 0.1]
	INS	0.51	0.22	[−0.3; 1.3]
	AMYG	−0.13	0.59	[−0.6; 1.3]
SCOPA-GI	PUT	−2.2	**0.01**	[−3.9; −0.5]
	CN	−1.48	**0.034**	[−2.9; −0.1]
	PAL	−0.06	0.90	[−1.1; 1]
	INS	−0.66	0.25	[−1.8; 0.5]
	AMYG	−0.2	0.54	[−0.8; 0.4]
SCOPA-URO	PUT	−2.7	**0.013**	[−4.6; −0.6]
	CN	−2.7	**0.002**	[−4.3; −1.0]
	PAL	−1.4	**0.025**	[−2.7; −0.2]
	INS	−0.66	0.36	[−2.1; 0.75]
	AMYG	−0.44	0.27	[−1.2; 0.4]
ESS	PUT	−0.79	0.63	[−4; 2.4]
	CN	−0.33	0.81	[−3.1; 2.4]
	PAL	−0.99	0.30	[−2.9; 0.9]
	INS	0.25	0.81	[−1.8; 2.3]
	AMYG	0.01	0.99	[−1.1; 1.1]
RBDSQ	PUT	−3.27	**0.010**	[−5.7; −0.8]
	CN	−2.35	**0.028**	[−4.4; − 0.3]
	PAL	−1.56	**0.036**	[−3.0; −0.1]
	INS	−0.08	0.92	[−1.7; 1.54]
	AMYG	0.37	0.40	[−0.52; 1.27]

Correlations between clinical scores and ^123^I-FP-CIT ROIs uptake. All uptake values are average (mean of left and right), except * = contralateral side to clinically more affected side. *PUT* putamen, *CN* caudate nucleus, *PAL* pallidum, *INS* insula, *AMYG* amygdala. Significant results are highlighted in bold

Regarding antidepressant medication, we observed that PD patients on SSRI/SNRI treatment had non-significantly lower ^123^I-FP-CIT uptake values in caudate nucleus, putamen, pallidum and insula compared to patients without antidepressants (4–8% reduction, all *p* > 0.11, *MWU*). Conversely, ^123^I-FP-CIT binding in the amygdala was slightly higher in patients on SSRI medication (9% increase, *p* = 0.10, *MWU*). SSRI/SNRI treatment was added as a dichotomised variable in the regression analyses and did not show any significant effect on striatal or extrastriatal uptake (all *p* > 0.10).

## Discussion

In the present work, we showed that in addition to a well-known decrease in ^123^I-FP-CIT uptake involving the striatum, early PD subjects exhibit a significant uptake reduction in extrastriatal regions including the pallidum, amygdala and insula. As extrastriatal ^123^I-FP-CIT binding mainly derives from SERT [2], we took advantage of the same scanning session of a large PD cohort to confirm early binding changes in regions outside the striatum compared to similarly-aged Controls.

^123^I-FP-CIT impairment in pallidum was recently observed by Lee et al. [21]. ^11^C-PE2I and ^11^C-DASB PET studies showed that while the lateral part of the pallidum (globus pallidum externus) has a similar proportion of serotonergic and dopaminergic terminals, the medial part (globus pallidus internus) is mainly a serotonergic nucleus [20]. In addition, our PD subjects exhibited an impaired extrastriatal uptake in the insula and amygdala. These findings confirm recent data by Pilotto et al. showing decreased ^123^I-FP-CIT uptake in insula, thalamus and cingulate in both PD and dementia with Lewy bodies [9]. Moreover, they confirm previous neuropathological evidence and in vivo ^11^C-DASB-PET and ^123^I-FP-CIT SPECT studies showing an altered serotonergic uptake for PD in the insula [6, 8], providing additional value for ^123^I-FP-CIT SPECT, a major diagnostic imaging tool in daily clinical practice. Findings of a reduced ^123^I-FP-CIT uptake in the amygdala are also in line with post-mortem studies using chromatography and enzyme-linked immunosorbent assay showing that PD subjects had reduced dopamine and noradrenaline levels in the amygdala [22].

Our correlation analysis confirmed that motor impairment as measured by MDS-UPDRS III total score was associated with decreased putaminal ^123^I-FP-CIT uptake [23]. It might seem surprising that MDS-UPDRS III was not more strongly correlated with striatal ROIs ^123^I-FP-CIT uptake. However, a recent psychometric assessment of MDS-UPDRS part II and III scales in PD subjects enrolled into the PPMI cohort showed an important floor effect, which can explain the moderate association for our cohort [24].

Clinical association with an extensive serotonergic deficit may include apathy, emotional disturbances, depression, cognitive deficits, and dysautonomic manifestations in PD, all of which being potentially present early in the disease course or even in its prodromal phase [25].

Previous studies did not show any significant correlation between striatal ^123^I-FP-CIT binding and non-motor symptoms such as fatigue, depression and excessive daytime sleepiness in PD [26]. Using a ROI-based approach to assess the contribution of extrastriatal ROIs, Qamhawi et al. observed a reduced ^123^I-FP-CIT binding in the raphe nucleus in early PD patients from the PPMI initiative compared to Controls (although only 12.5% of PD had values below 1.5 standard deviation). They also found that raphe nucleus binding was associated with rest tremor amplitude, constancy and severity, but not with non-motor symptoms such as depression, REM-sleep behaviour disorder and excessive daytime sleepiness [27].

While a trend association was found between GDS and pallidal ^123^I-FP-CIT uptake in the present study, we did not find a significant correlation between SERT-rich regional uptake and cognitive impairment as measured with MOCA. One reason might be that other monoaminergic pathways are involved in the pathophysiology of cognitive impairment [23] and that larger samples would be necessary to tackle such complex processes. In addition, our PD cohort mainly consisted of early subjects with relatively preserved cognition (mean MoCA 27.4), so admittedly, a significant correlation between SERT binding and depression/cognitive scales could have been observed in cohorts with a broader range of impairment [28].

Interestingly, correlations between striatal and extrastriatal regions binding among PD patients showed that caudate nucleus and putamen ^123^I-FP-CIT uptake impairment was associated with concomitant reductions in insula and amygdala binding in the full cohort analyses and also in the PD subgroup with GDS ≥ 5. However, when assessing these potential associations in the subgroup with mild cognitive impairment (MOCA ≤25, *n* = 30), we did not observe such correlations, except for a trend association between putamen and amygdala uptake (*p* = 0.09). The smaller subgroup could explain the lack of significant association. In addition, changes in striatal and extrastriatal binding can be of a different magnitude in patients with cognitive impairment, although we did not find any significant relationship between extrastriatal binding and cognitive impairment.

Our early PD group did not show any major signs of excessive daytime sleepiness, with ESS scores similar to Controls (*p* = 0.68). At variance with Yousaf et al. [29], no significant correlation between ESS and striatal ^123^I-FP-CIT uptake was observed in PD (all *p* > 0.29). Differences in the inclusion criteria could explain the apparently opposite findings. In fact, Yousaf et al. [29] performed between-group comparisons of PD with or without excessive daytime sleepiness, whereas we assessed correlations with striatal/extrastriatal ^123^I-FP-CIT using a cohort with relatively low ESS score.

In contrast, we observed that PD group had a significantly increased RBDSQ score (*p* < 0.001) relative to Controls, with 51/154 PD (33.1%) having RBDSQ ≥5. Moreover, higher RBDSQ was associated with lower ^123^I-FP-CIT binding in caudate nucleus, putamen and pallidum (all *p* < 0.05). These findings are in line with Pagano et al., who showed that RBD was associated with faster striatal dopaminergic decline [30].

Finally, a significant association was found between SCOPA-GI subscore and caudate/putamen uptake. This confirms previous observations from Hinkle et al. [31], who described an association between SCOPA-GI score (especially constipation items) and both regions. Additionally, we observed that the SCOPA-URO subscore was also negatively correlated with striatal uptake. This is in line with previous findings from Kim et al. [32] who recently found a correlation between putamen uptake and SCOPA-URO subscore also including the PPMI cohort. For both studies from Hinkle and Kim, SPECT/MRI coregistration was not performed. While our findings are of interest, they should be interpreted with an important caveat in mind. In fact, while bowel motility in late-life has been associated with postmortem neuron density in the substantia nigra [33], a causal relationship between presynaptic striatal dopamine uptake and gastrointestinal impairment in early PD would be hasty. Indeed, one hypothesis would be that dysautonomia would appear concurrently to striatal dopaminergic degeneration and not necessarily be driven by it.

Studies on rats and humans have shown conflicting results regarding the effect of antidepressant medication (SSRI/SNRI) on ^123^I-FP-CIT uptake values. In rats, sub-chronic administration of fluvoxamine was not associated with significant changes in striatal ^123^I-FP-CIT uptake [34]. In humans, higher binding in striatal regions and lower binding in extrastriatal regions was observed after acute treatment of paroxetine [35], while chronic treatment was associated with lower striatal binding [36], which is in line with our findings.

The present study has several major strengths: first, it is based on a large cohort of well-characterized PD and CTL subjects who underwent extensive clinical motor and non-motor evaluation. In addition, we included subjects whose SPECT was acquired within 3 months of a high-resolution anatomical MRI in order to proceed to MRI/SPECT coregistration and to provide PVE-corrected results. Although our included subjects represent about half of the total PD subjects in the PPMI cohort, given the stringent inclusion criteria we applied for analyses purposes, we believe this provides a major insight into the pathophysiology of monoaminergic degeneration in PD. The present work also presents some limitations. As this is the case in similar clinical studies, diagnoses are not based on neuropathology, so we cannot exclude diagnostic misattribution, especially since some PD cases were enrolled at a very early stage (10 subjects with < 6-month disease duration). In addition, SPECT acquisition was performed 3-4 h after ^123^I-FP CIT injection, which is the ideal timeframe for DAT evaluation, whereas the recommended time window for extrastriatal SERT is 2-3 h [2]. Nonetheless, due to a slow ^123^I-FP-CIT washout, we expect SERT binding to be relatively stable at 3-4 h [37].

## Conclusion

We presented evidence of a widespread ^123^I-FP-CIT extrastriatal impairment in early PD, spanning the amygdala and insular region, which is in keeping with neuropathological and ^11^C-DASB PET imaging. As ^123^I-FP-CIT SPECT imaging is used daily to confirm nigrostriatal degeneration, these results bring novel evidence for a potential role in helping to discriminate PD from non-degenerative conditions by assessing extrastriatal uptake. Further studies are warranted to assess whether atypical parkinsonian syndromes harbour similar extrastriatal ^123^I-FP-CIT impairment, therefore confirming complex monoamine transmission abnormalities in degenerative parkinsonisms.

## Data Availability

Anonymised imaging data analyses performed by the authors are available to researchers upon reasonable request.

## Abbreviations

^123^I-FP-CIT: ^123^I-N-ω-fluoropropyl-2β-carbomethoxy-3β-(4-iodophenyl) nortropane
SPECT: Single photon emission computed tomography
SERT: Serotonin transporter
^11^C-DASB: ^11^C-3-amino-4-(2-dimethylaminomethyl-phenylsulfanyl)benzonitrile
PET: Positron emission tomography
PD: Parkinson’s disease
PSP: Progressive supranuclear palsy (PSP);
ROI: Region of interest
MDS: Movement Disorders Society
UPDRS: Unified Parkinson’s disease rating scale
GDS: Geriatric Depression Scale
MoCA: Montreal Cognitive Assessment
SCOPA-AUT: Autonomic scale for outcome in Parkinson’s disease
ESS: Epworth Sleepiness Scale
RBDSQ: REM sleep behaviour disorder screening questionnaire
PPMI: Parkinson’s Progression Markers initiative
CTL: Controls
MPRAGE: Magnetization prepared rapid gradient echo
SPM: Statistical parametric mapping
FWE: Family-wise error
PVE: Partial volume effect
FWHM: Full-width at half maximum
REF: Reference
SBR: Striatal binding ratio
MWU: Mann-Whitney U test
VX: Voxels
CI: Confidence interval

## References

[1] Nicastro N, Garibotto V, Burkhard PR (2018). The role of molecular imaging in assessing degenerative parkinsonism - an updated review. Swiss Med Wkly.

[2] Koch W, Unterrainer M, Xiong G, Bartenstein P, Diemling M, Varrone A (2014). Extrastriatal binding of [(1)(2)(3)I]FP-CIT in the thalamus and pons: gender and age dependencies assessed in a European multicentre database of healthy controls. Eur J Nucl Med Mol Imaging.

[3] Hornung JP (2003). The human raphe nuclei and the serotonergic system. J Chem Neuroanat.

[4] Kerenyi L, Ricaurte GA, Schretlen DJ, McCann U, Varga J, Mathews WB (2003). Positron emission tomography of striatal serotonin transporters in Parkinson disease. Arch Neurol.

[5] Kish SJ, Tong J, Hornykiewicz O, Rajput A, Chang LJ, Guttman M (2008). Preferential loss of serotonin markers in caudate versus putamen in Parkinson's disease. Brain..

[6] Guttman M, Boileau I, Warsh J, Saint-Cyr JA, Ginovart N, McCluskey T (2007). Brain serotonin transporter binding in non-depressed patients with Parkinson's disease. Eur J Neurol.

[7] Kaasinen V, Joutsa J, Noponen T, Johansson J, Seppanen M (2015). Effects of aging and gender on striatal and extrastriatal [123I]FP-CIT binding in Parkinson's disease. Neurobiol Aging.

[8] Premi E, Calhoun VD, Garibotto V, Turrone R, Alberici A, Cottini E (2017). Source-based Morphometry multivariate approach to analyze [(123) I]FP-CIT SPECT imaging. Mol Imaging Biol.

[9] Pilotto A, Schiano di Cola F, Premi E, Grasso R, Turrone R, Gipponi S (2019). Extrastriatal dopaminergic and serotonergic pathways in Parkinson's disease and in dementia with Lewy bodies: a (123) I-FP-CIT SPECT study. Eur J Nucl Med Mol Imag.

[10] Parkinson Progression Marker I (2011). The Parkinson Progression Marker Initiative (PPMI). Prog Neurobiol.

[11] Goetz CG, Tilley BC, Shaftman SR, Stebbins GT, Fahn S, Martinez-Martin P (2008). Movement Disorder Society-sponsored revision of the unified Parkinson's disease rating scale (MDS-UPDRS): scale presentation and clinimetric testing results. Movement Dis.

[12] Yesavage JA, Brink TL, Rose TL, Lum O, Huang V, Adey M (1982). Development and validation of a geriatric depression screening scale: a preliminary report. J Psychiatr Res.

[13] Nasreddine ZS, Phillips NA, Bedirian V, Charbonneau S, Whitehead V, Collin I (2005). The Montreal cognitive assessment, MoCA: a brief screening tool for mild cognitive impairment. J Am Geriatr Soc.

[14] Visser M, Marinus J, Stiggelbout AM, Van Hilten JJ (2004). Assessment of autonomic dysfunction in Parkinson's disease: the SCOPA-AUT. Movement Dis.

[15] Johns MW (1991). A new method for measuring daytime sleepiness: the Epworth sleepiness scale. Sleep..

[16] Stiasny-Kolster K, Mayer G, Schafer S, Moller JC, Heinzel-Gutenbrunner M, Oertel WH (2007). The REM sleep behavior disorder screening questionnaire--a new diagnostic instrument. Movement Dis.

[17] Hoehn MM, Yahr MD (1967). Parkinsonism: onset, progression and mortality. Neurology..

[18] Gonzalez-Escamilla G, Lange C, Teipel S, Buchert R, Grothe MJ (2017). Alzheimer's disease neuroimaging I. PETPVE12: an SPM toolbox for partial volume effects correction in brain PET - application to amyloid imaging with AV45-PET. Neuroimage..

[19] Desikan RS, Segonne F, Fischl B, Quinn BT, Dickerson BC, Blacker D (2006). An automated labeling system for subdividing the human cerebral cortex on MRI scans into gyral based regions of interest. Neuroimage..

[20] Beaudoin-Gobert M, Epinat J, Metereau E, Duperrier S, Neumane S, Ballanger B (2015). Behavioural impact of a double dopaminergic and serotonergic lesion in the non-human primate. Brain..

[21] Lee JY, Lao-Kaim NP, Pasquini J, Deuschl G, Pavese N, Piccini P (2018). Pallidal dopaminergic denervation and rest tremor in early Parkinson's disease: PPMI cohort analysis. Parkinsonism Relat Disord.

[22] Buddhala C, Loftin SK, Kuley BM, Cairns NJ, Campbell MC, Perlmutter JS (2015). Dopaminergic, serotonergic, and noradrenergic deficits in Parkinson disease. Ann Clin Transl Neurol.

[23] Pirker W, Holler I, Gerschlager W, Asenbaum S, Zettinig G, Brücke T (2003). Measuring the rate of progression of Parkinson's disease over a 5-year period with beta-CIT SPECT. Movement Dis.

[24] Regnault A, Boroojerdi B, Meunier J, Bani M, Morel T, Cano S (2019). Does the MDS-UPDRS provide the precision to assess progression in early Parkinson's disease? Learnings from the Parkinson's progression marker initiative cohort. J Neurol.

[25] Maillet A, Krack P, Lhommee E, Metereau E, Klinger H, Favre E (2016). The prominent role of serotonergic degeneration in apathy, anxiety and depression in de novo Parkinson's disease. Brain..

[26] Park SB, Kwon KY, Lee JY, Im K, Sunwoo JS, Lee KB (2019). Lack of association between dopamine transporter loss and non-motor symptoms in patients with Parkinson's disease: a detailed PET analysis of 12 striatal subregions. Neurol Sci.

[27] Qamhawi Z, Towey D, Shah B, Pagano G, Seibyl J, Marek K (2015). Clinical correlates of raphe serotonergic dysfunction in early Parkinson's disease. Brain..

[28] Schrag A, Siddiqui UF, Anastasiou Z, Weintraub D, Schott JM (2017). Clinical variables and biomarkers in prediction of cognitive impairment in patients with newly diagnosed Parkinson's disease: a cohort study. Lancet Neurol.

[29] Yousaf T, Pagano G, Niccolini F, Politis M (2018). Excessive daytime sleepiness may be associated with caudate denervation in Parkinson disease. J Neurol Sci.

[30] Pagano G, De Micco R, Yousaf T, Wilson H, Chandra A, Politis M (2018). REM behavior disorder predicts motor progression and cognitive decline in Parkinson disease. Neurology..

[31] Hinkle JT, Perepezko K, Mills KA, Mari Z, Butala A, Dawson TM (2018). Dopamine transporter availability reflects gastrointestinal dysautonomia in early Parkinson disease. Parkinsonism Relat Disord.

[32] Kim R, Jun JS (2019). Association of autonomic symptoms with presynaptic striatal dopamine depletion in drug-naive Parkinson's disease: an analysis of the PPMI data. Auton Neurosci.

[33] Petrovitch H, Abbott RD, Ross GW, Nelson J, Masaki KH, Tanner CM (2009). Bowel movement frequency in late-life and substantia nigra neuron density at death. Movement Dis.

[34] Lavalaye J, Knol RJ, de Bruin K, Reneman L, Janssen AG, Booij J. [123I]FP-CIT binding in rat brain after acute and sub-chronic administration of dopaminergic medication. Eur J Nucl Med. 2000;27 3:346–9; doi: 10.1007/s002590050044. 10.1007/s002590050044 10774889

[35] Booij J, de Jong J, de Bruin K, Knol R, de Win MM, van Eck-Smit BL (2007). Quantification of striatal dopamine transporters with 123I-FP-CIT SPECT is influenced by the selective serotonin reuptake inhibitor paroxetine: a double-blind, placebo-controlled, crossover study in healthy control subjects. J Nuclear Med.

[36] Rominger A, Cumming P, Brendel M, Xiong G, Zach C, Karch S (2015). Altered serotonin and dopamine transporter availabilities in brain of depressed patients upon treatment with escitalopram: a [123 I]beta-CIT SPECT study. Eur Neuropsychopharmacol.

[37] Abi-Dargham A, Gandelman MS, DeErausquin GA, Zea-Ponce Y, Zoghbi SS, Baldwin RM (1996). SPECT imaging of dopamine transporters in human brain with iodine-123-fluoroalkyl analogs of beta-CIT. J Nuclear Med.

